# Efficacy of a new ready-to-use PCV2 and *Mycoplasma hyopneumoniae* vaccine under field conditions

**DOI:** 10.17221/25/2025-VETMED

**Published:** 2025-11-27

**Authors:** Peter Trampus, Attila Csagola, Tamas Szalai, Nimrod Palmai, Adam Toth, Nora Terenyi, Zoltan Nagy, Nikoletta-Agnes Szeplaki, Gergely Somogyi, Eniko Rausch, Zoltan Penzes, Roman Krejci

**Affiliations:** ^1^Ceva-Phylaxia Zrt., Budapest, Hungary; ^2^Ceva Sante Animale, Libourne Cedex, France

**Keywords:** bivalent, immunisation, PCV2d, PRDC, swine, trial

## Abstract

Coinfection of porcine circovirus type 2 (PCV2) with *Mycoplasma hyopneumoniae* (*M. hyo*) causes major worldwide economic losses within the swine industry. This study aimed to assess the safety and efficacy of a single dose of a bivalent vaccine containing PCV2d and *M. hyo* antigen (Cirbloc^®^ M Hyo) under field conditions. Two studies were performed under the GCP (Good Clinical Practice) requirements on farrow-to-finish farms in Hungary and Cyprus. On both farms, the presence of both PCV2 and *M. hyo* infection was demonstrated. For both studies, safety parameters were observed and measured from inclusion at 21 (±3) days of age until 14 days after vaccination. Efficacy parameters were observed and measured from inclusion until slaughter. Administration of the vaccine was safe in both studies, as no general, immediate, or local reactions were observed. The efficacy of the vaccine was confirmed in both studies as the following parameters were significantly reduced in the vaccinated groups compared to the control groups: viraemia, faecal shedding, viral load in lungs and in all collected lymphoid tissues, *M. hyo*-specific lung lesions, and average daily body weight gain. These results collectively support the vaccine’s potential as an effective tool for disease control.

*Mycoplasma hyopneumoniae* (*M. hyo*) and porcine circovirus type 2 (PCV2) are two pathogens that play a significant role in the development of the porcine respiratory disease complex (PRDC), resulting in substantial economic losses in global swine production (Chae 2016; Yang et al. 2020; Eddicks et al. 2021). PRDC is a multifactorial disease characterised by respiratory signs, reduced growth performance, and decreased feed efficiency (Halbur 1996; Thacker 2001; Eddicks et al. 2021).

PCV2 is a member of the family *Circoviridae* containing a circular, single-stranded DNA genome (Segales 2012). The first disease associated with a PCV2 infection was the post-weaning multisystemic wasting syndrome (PMWS), reported in Canada in 1996 (Clark 1996; Harding 1996).

Subsequently, PCV2 has been associated with several pathological conditions collectively referred to as porcine circovirus diseases (PCVD) (Allan et al. 2002) or porcine circovirus-associated diseases (PCVAD) (Harding 2007).

PCV2 is divided into at least eight genotypes, including PCV2a, PCV2b, and PCV2d, that are considered the major genotypes (Guo et al. 2022). PCV2d has been showing an increasing prevalence compared to other genotypes (Xiao et al. 2015; Franzo and Segales 2020). *M. hyo*, a bacterium with a small genome lacking a cell wall (pleomorphic), is the primary pathogen of enzootic pneumonia (EP) (Thacker and Minion 2012).

This microorganism can mainly be found on the mucosal surface of the trachea, bronchi, and bronchioles, disrupting the cilia on the epithelial surface (Blanchard et al. 1992). It can also alter the immune system of the respiratory tract, making it more susceptible to concurrent infections with other respiratory pathogens, including bacteria, parasites, and viruses (Thacker and Minion 2012).

As PRDC significantly impacts swine production, the development of efficacious vaccines for prevention is highly important. A bivalent vaccine with one-dose administration providing immunity against both PCV2 and *M. hyo* offers several advantages. Besides reduced vaccine costs, fewer interventions generate less stress for the animals (Lopez-Lorenzo et al. 2021; Yang et al. 2021; Sipos and Sipos 2022).

Most of these vaccines are the PCV2a or PCV2b genotypes combined with the *M. hyo* antigen. As PCV2d is the most frequent genotype, there is a great demand for vaccines offering protection against infection by this PCV2 strain (Xiao et al. 2015; Xiao et al. 2016). Cirbloc^®^ M Hyo, a new vaccine combining the PCV2d and *M. hyo* antigen in the ready-to-use form, was successfully tested under experimental conditions.

This article demonstrates the safety and efficacy under field conditions.

## MATERIAL AND METHODS

### Farm history and study design

Two studies were performed following GCP (Good Clinical Practice) standards in two farrow-to-finish commercial farms in Hungary and Cyprus.

The farms were selected based on the following criteria: the presence of PCV2 and *M. hyo* infection, and the absence of porcine reproductive and respiratory syndrome (PRRS) infection.

On the Hungarian farm (Farm No. 1), the animals had not received vaccination before inclusion in the study, and the non-study animals were regularly immunised with a commercial vaccine against PCV2 and *M. hyo.* On the farm in Cyprus (Farm No. 2), none of the animals, including the non-study animals, were vaccinated against PCV2 and *M. hyo*.

On each farm, one group of 3-week-old piglets (Farm No. 1: *n* = 116; Farm No. 2: *n* = 318) was vaccinated with Cirbloc^®^ M Hyo and compared to a group of piglets (Farm No. 1: *n* = 117; Farm No. 2: *n* = 317) treated with phosphate-buffered saline (PBS) as a control product. For assessing vaccine safety, focus groups were established as follows: 30–30 piglets on Farm No. 1 and 28–28 piglets on Farm No. 2 were selected from each treatment group. The vaccine or the PBS (2 ml of each) was administered intramuscularly (i.m.) on the left side of the neck at 21 days of age. The study was conducted blindly; the piglets of both groups were randomised to be similar in age, maternal origin, body weight, and sex ratio. The pigs involved in the study were fed the same feed as all other standard production pigs on the farm, which fulfils the physiological needs of the animals in quality and quantity.

### Safety evaluation

In both trials, safety parameters were observed and measured from inclusion at 21 (±3) days of age until 14 days (D14) after vaccination. All included animals were observed for mortality, morbidity, and adverse events, and monitored for immediate reactions following vaccination. Rectal temperature was measured in the focus groups the day before vaccination (D–1), just prior to vaccination (D0h0), 4 h (D0h4), and on four consecutive days after vaccination (D1–D4). The general health status of the piglets and local reactions at the injection site were checked individually in the focus groups on D–1, D0, D0h4, and then daily from D1 to D14. Weight gain was measured by individual weighing of the piglets in the focus group at inclusion (D–1), D7 (Farm No. 1), and D14.

### Efficacy evaluation

In both studies, efficacy parameters were observed and measured from inclusion at 21 (±3) days of age until slaughter. To assess PCV2 viraemia, blood samples were taken from the animals in the focus group at inclusion (D–1) and further in the 4^th^, 7^th^, 10^th^, 15^th^, 18^th^, and 21^st^ weeks of the study.

PCV2 viral load of serum samples was determined by real-time quantitative PCR (Brunborg et al. 2004). To determine PCV2 shedding, faecal swab samples were taken from the focus group animals at inclusion (D–1) and again in the 4^th^, 7^th^, 10^th^, 15^th^, 18^th^, and 21^st^ weeks of the study. To define PCV2 viral load, lung and selected lymphoid tissue samples were collected from 45 pigs per treatment group (Farm No. 1) and 58 vaccinated and 54 control animals (Farm No. 2) at slaughter.

All piglets were weighed individually at inclusion, 14 days following vaccination, at the end of nursery (Farm No. 1), and at the end of fattening.

Lung scoring was performed on the lungs of pigs from the vaccinated group (Farm No. 1: *n* = 102; Farm No. 2: *n* = 229) and from the control group (Farm No. 1: *n* = 98; Farm No. 2: *n* = 218) at slaughter. Lung lesions were described as the estimated proportion (%) of consolidated (grey reddish) area per lobe, and the percentages were transferred to scores. Weighted lung score values were calculated according to the description in the monograph of European Pharmacopoeia (Ph. Eur.) 2448 “Porcine Enzootic Pneumonia vaccine (Inactivated)” in force.

### Statistical analysis

In the statistical analysis, the different kinds of parameters were analysed differently. Chi-square tests were applied for categorical variables such as mortality and morbidity. Continuous variables were compared between the groups (after log10 transformation if necessary) with the Welch test or linear mixed model with least square means, regarding the complexity of the parameter (if it is not marked in the results section, the mixed model was used).

Results of all statistical hypothesis tests were considered significant if *P* < 0.05. The presented statistical calculations (*P*-values) were performed using SAS v9.4 (SAS Institute Inc., USA) or Stata v17 software (StataCorp LLC, USA).

## RESULTS

### Morbidity, mortality, immediate-, local-, and general reactions and adverse events

No adverse event, immediate reaction, or general and local reaction occurred in any of the trials.

On Farm No.1, neither morbidity nor mortality occurred during the safety period. On Farm No. 2, the difference in morbidity was not significant between the vaccinated and the control group, neither in the safety observation period (chi-square test *P** = ***0.751 6, Fisher’s two-sided *P** = ***1.000) nor during the whole study (chi-square test *P** = ***0.562 6, Fisher’s two-sided *P** = ***0.575 4).

Six mortality cases occurred on this farm during the safety observation period (3 in each group), and none were associated with the vaccination. The causes of death were infection with *Glaesserella* (Haemophilus) *parasuis* (*n* = 3), post-weaning diarrhoea (*n* = 3), crushing by a sow (*n* = 1), and from an unknown origin in one animal.

### Rectal temperature

In both trials, the individual maximum increase of temperature (iMIT) after treatment was higher in the vaccinated groups 4 h after vaccination (Farm No. 1: 0.75 °C on average; Farm No. 2: 0.77 °C on average) but returned to the baseline 24 h after treatment. Both values meet the requirements defined in the Ph. Eur.

### PCV2 viraemia

**Figure 1:**
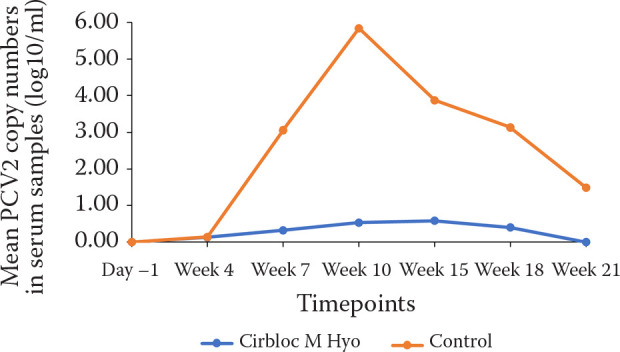
Viraemia in pigs at different timepoints on Farm No. 1 PCV2 = porcine circovirus type 2

**Figure 2:**
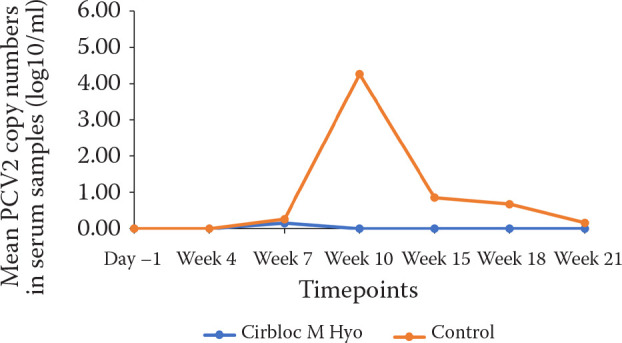
Viraemia in pigs at different timepoints on Farm No. 2 PCV2 = porcine circovirus type 2

PCV2 viraemia was significantly reduced in the vaccinated group compared to the control group from week 7 to week 21 (Farm No. 1) and from week 10 to week 18 (Farm No. 2), as shown in Figures 1 and 2. The *P*-values are as follows:

Farm No. 1: in week 7 (*P* = 0.000 1), in week 10 (*P* = 0.000 1), in week 15 (*P* = 0.000 1), in week 18 (*P* = 0.000 1), and in week 21 (*P* < 0.001 7). The maximum reduction was log10 5.30 at week 10.

Farm No. 2: in week 10 (*P* < 0.000 1), in week 15 (*P* = 0.002 3), and in week 18 (*P* = 0.016 6). The maximum reduction was log10 4.26 at week 10.

### Faecal shedding of PCV2

The shedding via faeces was significantly reduced in the vaccinated group compared to the control group for Farm No. 1 in week 7 (*P* = 0.024 4) and for Farm No. 2 in weeks 10 (*P* = 0.009) and 18 (*P* < 0.000 1).

### PCV2 load

**Figure 3:**
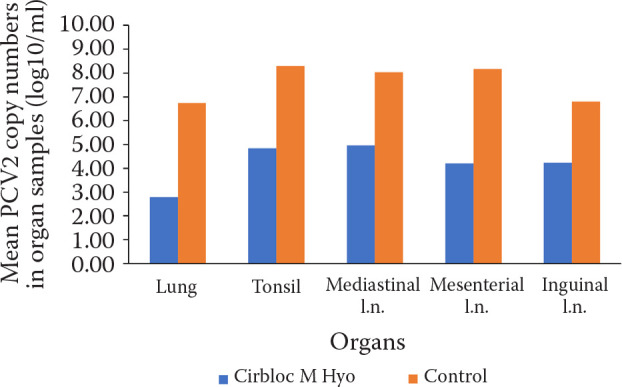
PCV2 viral load in organ samples of pigs on Farm No. 1 l.n. = lymph node; PCV2 = porcine circovirus type 2

**Figure 4:**
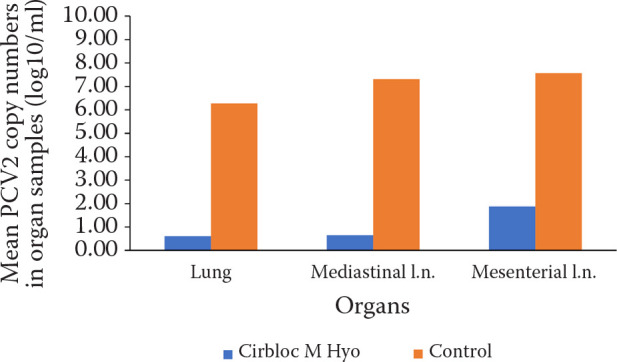
PCV2 viral load in organ samples of pigs on Farm No. 2 l.n. = lymph node; PCV2 = porcine circovirus type 2

The PCV2 load was significantly reduced in lung samples and in all collected lymphoid tissues of the vaccinated animals at slaughter in both studies (*P* < 0.000 1 for all samples), as shown in Figures 3 and 4. Sampling of tonsils and inguinal lymph nodes from animals of Farm No. 2 was not performed for safety reasons. Tonsils and inguinal lymph nodes remained in the carcasses on the slaughter line because removing them would have been associated with a high risk of injury.

PCV2b genotype was identified on Farm No. 1, while PCV2d circulated on Farm No. 2.

### Lung lesion scoring

**Figure 5:**
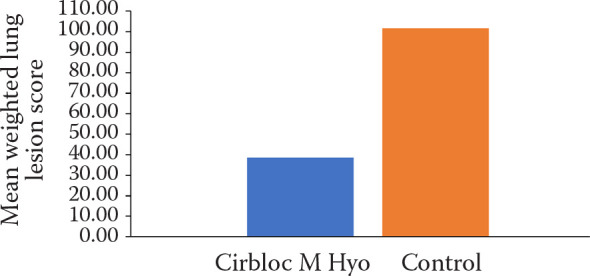
Mean weighted lung lesion scores (LLS) of the groups on Farm No. 1

**Figure 6:**
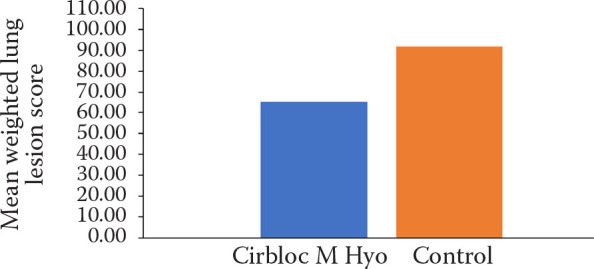
Mean weighted lung lesion scores (LLS) of the groups on Farm No. 2

As indicated in Figures 5 and 6, the vaccinated group’s mean lung lesion score (LLS) was significantly reduced on both farms compared to the control group (*P* = 0.000 1, Welch test, respectively).

### Average daily weight gain

**Figure 7:**
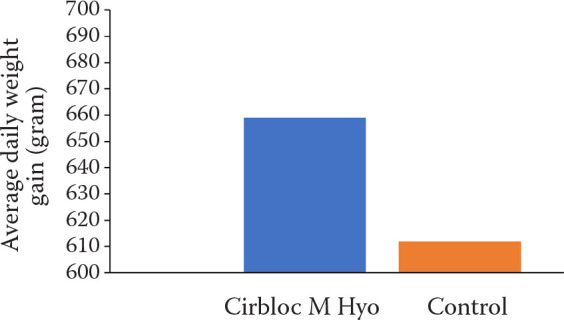
Average daily weight gain (ADWG) of pigs on Farm No. 1

**Figure 8:**
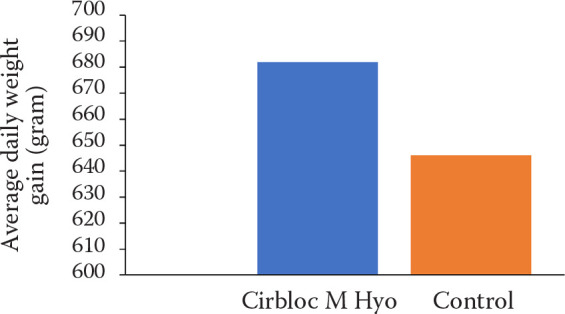
Average daily weight gain (ADWG) of pigs on Farm No. 2

In both trials, average daily weight gain (ADWG) was found significantly higher in the vaccinated animals than in the controls [Farm No. 1: 47 g difference (659 g vs 612 g), *P* = 0.000 1, Welch test; Farm No. 2: 36 g difference (682 g vs 646 g, *P* < 0.000 1)] as demonstrated in Figures 7 and 8.

## DISCUSSION

PCV2 and *M. hyo* are pathogens that have a major impact on swine health and industry. Piglet vaccination is one of the most important tools for achieving protection against PCV2 and *M. hyo-*related diseases. Due to PCV2’s high genetic variability, new prevalent genotypes, and other aspects, including animal welfare and cost-effectiveness, vaccine development constantly faces multiple challenges (Yang et al. 2021; Guo et al. 2022; Sipos and Sipos 2022).

The tested vaccine is intended for the active immunisation of pigs to reduce viraemia, viral load in the lungs and lymphoid tissues, virus shedding caused by PCV2 infection, and severity of lung lesions caused by *M. hyo* infection. In previous trials, the efficacy of a new ready-to-use PCV2 and *M. hyo* vaccine (Cirbloc^®^ M Hyo) was demonstrated under experimental conditions (pre-clinical studies). Two trials were conducted on farrow-to-finish farms in Hungary and Cyprus, where both pathogens were present, to evaluate the safety and field efficacy of this vaccine.

The PCV2 epidemiology differed between the two farms. Different PCV2 genotypes were present: PCV2b on Farm No. 1 and PCV2d on Farm No. 2; and the onset and duration of PCV2 circulation also differed. Pigs in the control group on Farm No. 1 were already infected between 4–7 weeks of age, and average viraemia was still close to 2 logs on week 21. In contrast, a single peak of short-lasting viraemia was found on week 10 on Farm No. 2. The patterns of *M. hyo* infections appeared similar in both farms regarding serological profile (data not shown) and final lung scoring results in the control groups. PCV2 infection dynamics, namely early and long-lasting PCV2 circulation, might be more important than genotype specificity and could be the reason behind better weight gains in vaccinated animals on Farm No. 1 (more severely affected by PCV2 infection) when comparing growth performance.

Administration of the vaccine was safe in both trials, as no adverse event, immediate reaction, or general or local reaction occurred. One study recorded some mortality cases during the safety period; however, those were not associated with the vaccination. Also, morbidity between the vaccinated and the control group was not significantly different. Although the individual rectal temperatures showed a temporary increase after treatment in both studies, they returned to the baseline 24 h after treatment.

For the efficacy parameters, viraemia, faecal shedding, viral load in all collected lymph node and lung samples (PCV2), and lung lesions (*M. hyo*) were significantly reduced in the vaccinated group compared to the non-vaccinated control group in both studies.

Looking at the data more closely, the reduction of PCV2 load in lung and lymphatic tissues appears to be stronger on Farm No. 2, whereas viral load in control animals is at similar levels. Also, the severity of lung lesions in control animals was similar on both farms; however, the reduction of lung lesions was more pronounced on Farm No. 1. There are several factors that might differ between the two farms and can impact the investigated parameters. Primarily, general health status and the presence of other pathogens might affect the susceptibility to PCV2 and *M. hyo*. In addition, numerous aspects might influence the immune system of the animals and the infection pressure, including feed and herd management, stress factors, infection control, and biosecurity measures. More data should be collected from both farms for a detailed comparison of these parameters, and a thorough review of these would be necessary to conclude the differences in results.

As reduced growth performance is a characteristic sign of PCVD and EP, average daily body weight gain (ADWG) was evaluated in both trials. It was found to be significantly higher in the vaccinated group than in the control group.

The significant difference in ADWG in pigs vaccinated against both PCV2 and *M. hyo* was reported previously (Tzika et al. 2015; Duivon et al. 2018), with even indicated differences in growth performance when different vaccines were used (Lopez-Lorenzo et al. 2021).

As demonstrated here, the results of the two field trials aligned with the outcomes of the previous laboratory studies. The vaccine was safe and protected against PCV2 and *M. hyo* infection when applied to piglets at 3 weeks of age under field conditions.

This novel vaccine, based on the most prevalent genotype PCV2d combined with *M. hyo* bacterin, proved to be highly efficient as a ready-to-use single-dose product for protecting against PCV2 and *M. hyo* infections.
